# Type 2 dendritic cells mediate control of cytotoxic T cell resistant tumors

**DOI:** 10.1172/jci.insight.145885

**Published:** 2021-09-08

**Authors:** Stephen Iwanowycz, Soo Ngoi, Yingqi Li, Megan Hill, Christopher Koivisto, Melodie Parrish, Beichu Guo, Zihai Li, Bei Liu

**Affiliations:** 1 Department of Microbiology and Immunology, Hollings Cancer Center, Medical University of South Carolina (MUSC), Charleston, South Carolina, USA.; 2 Division of Hematology, Department of Internal Medicine, The Ohio State University Comprehensive Cancer Center, Columbus, Ohio, USA.; 3 Department of Biochemistry and Molecular Biology, MUSC, Charleston, South Carolina, USA.; 4 Division of Medical Oncology and; 5 The Pelotonia Institute for Immuno-Oncology, The Ohio State University Comprehensive Cancer Center, Columbus, Ohio, USA.

**Keywords:** Immunology, Breast cancer, Chaperones, Dendritic cells

## Abstract

Type 2 DCs (DC2s) comprise the majority of conventional DCs within most tumors; however, little is known about their ability to initiate and sustain antitumor immunity, as most studies have focused on antigen cross-presenting DC1s. Here, we report that DC2 infiltration identified by analysis of multiple human cancer data sets showed a significant correlation with survival across multiple human cancers, with the benefit being seen in tumors resistant to cytotoxic T cell control. Characterization of DC subtype infiltration into an immunotherapy-resistant model of breast cancer revealed that impairment of DC1s through 2 unique models resulted in enhanced DC2 functionality and improved tumor control. *BATF3* deficiency depleted intratumoral DC1s, which led to increased DC2 lymph node migration and CD4^+^ T cell activation. Enhancing DC2 stimulatory potential by genetic deletion of *Hsp90b1* (encoding molecular chaperon GP96) led to a similar enhancement of T cell immunity and improved survival in a spontaneous breast cancer model. These data highlight the therapeutic and prognostic potential of DC2s within checkpoint blockade–resistant tumors.

## Introduction

Breakthroughs in understanding the roles of immune cells as active members of the complex tumor microenvironment have led to the development of revolutionary immunotherapies capable of inducing sustained remission in numerous cancer types (1). Prime examples include the recently FDA-approved immune checkpoint blockade (ICB) and chimeric antigen receptor (CAR) T cell therapies that unleash potent cytotoxic T cells on tumors. In spite of their potential, favorable outcomes are currently achieved in only a minority of patients (2–5), underscoring the need to better understand the complex interactions between infiltrating immune cells and the tumor microenvironment.

Tumor mass consists of highly heterogeneous transformed cells that survived various levels of immunological stress, which is dependent on their tissue of origin and mutational load (6–8). Cancer cells develop equally heterogeneous means to avoid immune clearance at multiple points during the tumor immunity cycle: tumor recognition, antigen trafficking, and effector cell activation/infiltration (9). Immunogenic tumors with a high mutation burden tend to interfere with end-stage T cell responses, thus requiring treatments that target checkpoints, such as PD-1 blockade therapy, in order to normalize immunity (2, 10, 11). Other tumors block the initiation of immunity through education and recruitment of tolerogenic immune cells, including tumor-associated macrophages (TAMs) and Tregs; the treatment of such tumors will require generating de novo immune responses (2). Hence, the characterization of the immunological signature of tumors is critical for identifying tumor-specific immune suppression and avoidance pathways. Breast cancer, in particular, has been shown to have highly heterogeneous immune environments (12–15). Clinical studies revealed that only a small subclass of breast cancer patients (Ki67^hi^, ER^+^) can be classified as immune enabled, with improved survival correlated to increased immune cell infiltration (14, 15). In general, breast cancer patients have a poor response to ICB, a finding that is reciprocated in mouse models (4, 16, 17). On the contrary, eliminating dominant suppressor cells, such as Tregs or TAMs, can reestablish control of breast tumors through the activation of immune effector cells (16, 18–20). Successful immunotherapy for breast cancer will require identifying and targeting suppressor cells, as well as the upstream events that control their activity.

Conventional DCs (cDCs) regulate the type, duration, and magnitude of adaptive immune responses (21). Tolerogenic or immunogenic responses can be induced by cDCs, depending on their surrounding environment and the nature of the antigen (22). cDCs develop from BM precursors as a separate lineage from other myeloid cell populations and differentiate into 2 subtypes with distinct functional specialization: cDC1 and cDC2 (23). Irf8- and Batf3-dependent cDC1 (Xcr1^+^CD103^+^) specialize in antigen cross-presentation with MHC class I (MHCI) molecules for activating CD8^+^ T cells, while Irf4-dependent cDC2 (CD11b^+^CD172^+^) primarily present antigens with MHCII for CD4^+^ T cell recognition and priming (24). Both cDC subtypes can also display strong tolerogenic properties, with either population capable of maintaining oral tolerance when the other is depleted (25). Within tumors, therapeutic activation of tumor-infiltrating DCs (tiDCs) has been shown to improve antitumor immunity significantly under homeostatic conditions; tiDCs are educated by many tumor types to adopt a regulatory phenotype (26–29). Little is known about the specific role of each DC subtype in different tumor microenvironments, and consequently, there have been few studies on how to target these cells specifically. cDC1s have been reported to be critical for the generation of an antitumor immune response against melanoma (30–32), yet they were shown to express a high level of inhibitory surface receptors in breast cancer (33). In comparison, cDC2s comprise a significantly larger portion of the tiDC population, but little is known about their functional properties, since these cells closely resemble monocyte-derived DCs, which are functionally and ontogenically distinct from cDCs (23). tiDC2s are primarily reported as suppressive (31, 34, 35); however, a recent study found them to be essential for generating antitumor CD4^+^ T cell responses (36). Altogether, the specific role of DC subtypes is likely to be tumor type dependent. Further characterization is needed to determine which DC subtype is most effective at generating antitumor immunity under different immune contextures.

In this study, we sought to identify the specific role of DC subtypes within tumors with different immune contextures. We investigated the prognostic value of DC subtype gene signatures within human tumors and found that DC2 infiltration displayed a stronger correlation with survival in breast cancer patients than DC1, particularly in highly suppressive subtypes. Mechanistic studies using a clinically relevant model of breast cancer revealed that, following depletion of DC1s, DC2s increased migration and significantly enhanced antitumor immunity. DC2 migration led to CD4^+^ T cell–dependent tumor control through the repolarization of TAMs toward an inflammatory M1-like phenotype. Furthermore, we discovered that enhancing DC2 stimulatory capacity by genetic deletion of chaperone GP96 promotes immune control of spontaneous breast cancer and significantly improves survival. Our results suggest that empowering the DC2–CD4^+^ T cell axis may hold promise for treating breast cancers insensitive to cytotoxic T cells.

## Results

### Immune context determines the impact of DC2 infiltration for human cancers.

**Figure 1:**
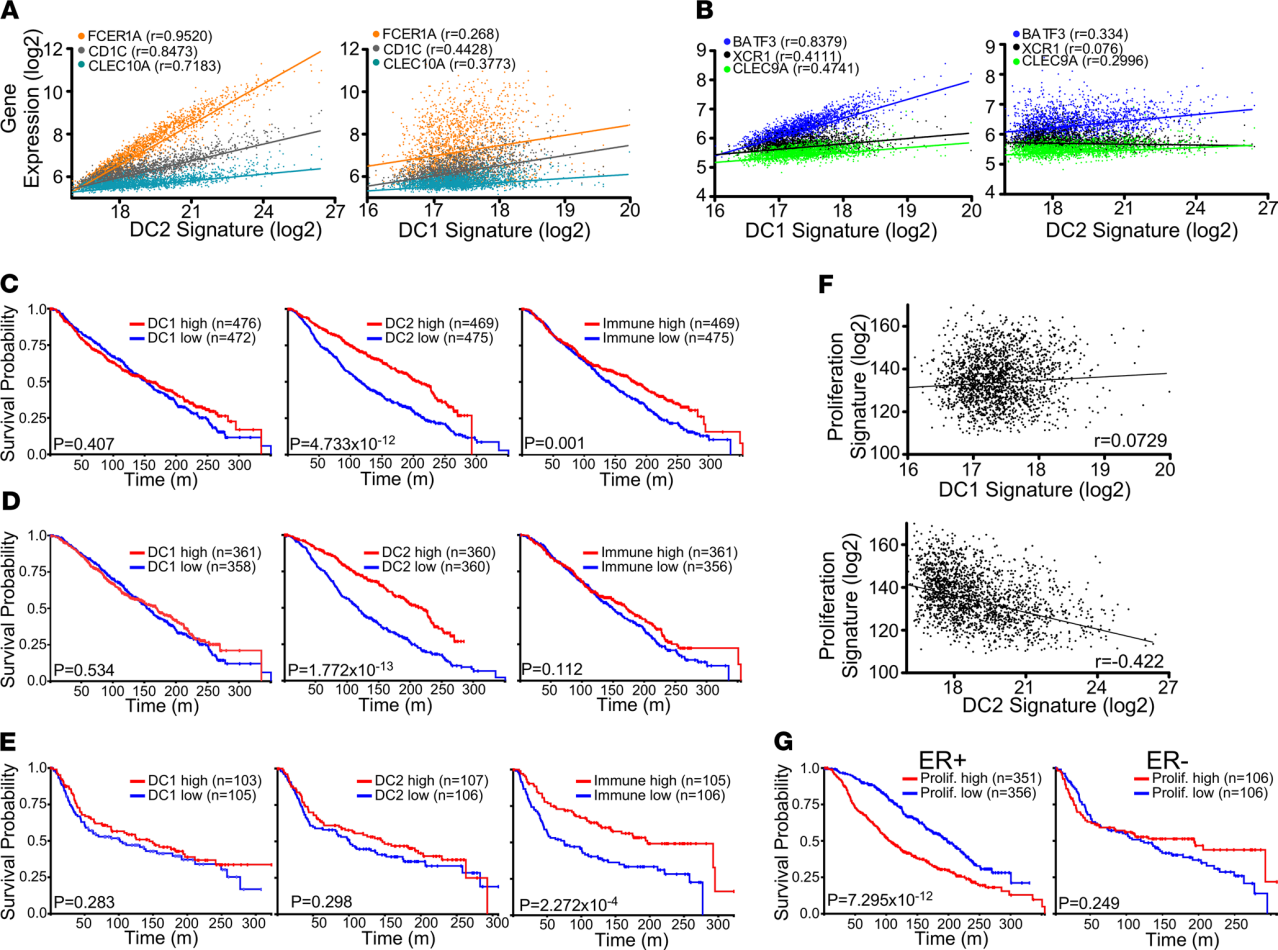
DC2 gene signature within the tumor correlates with improved survival in human breast cancer patients. (**A**) Correlation of genes that comprise the DC2 signature with the signatures for DC2 (left) and DC1 (right). (**B**) Correlation of genes that comprise the DC1 signature with the signatures for DC1 (left) and DC2s (right). A total of 1904 breast tumors from the METABRIC data set were used for analysis. (**C**) Kaplan-Meier curves for the overall survival of patients within the top or bottom quartile for the indicated gene signatures. (**D**) Kaplan-Meier curves for overall survival of patients with ER^+^ tumors within the top or bottom quartile for the indicated gene signatures. (**E**) Kaplan-Meier curves for overall survival of patients with ER^–^ tumors within the top or bottom quartile for the indicated gene signatures. (**F**) Correlation of proliferation gene signature with the signatures for DC1 (top) and DC2s (bottom). (**G**) Kaplan-Meier curves for overall survival of patients with ER^+^ or ER^–^ tumors within the top or bottom quartile for the gene signatures of proliferation. (**C**, **D**, **E**, and **G** were analyzed by a log-rank test; **F** was analyzed using Pearson’s correlation).****

DC subtypes have been reported to be strong predictors of disease outcome in multiple cancer types (37–39). However, the microenvironment of breast cancer is highly heterogeneous, with the prognostic value of immunity genes depending on estrogen receptor (ER) expression and proliferation status (15). We developed a DC2 gene signature (Figure 1A) based on genes identified to be uniquely expressed in DC2s by Villani et al. (40). We compared the DC2 signature and a DC1 signature (Figure 1B, adapted from ref. 37) to a published Immune response signature (15) in order to identify the prognostic value of tiDC composition compared with general immune infiltration. We analyzed primary tumor samples from the publicly available data set METABRIC (https://github.com/cBioPortal/datahub/tree/ab7cb6294676e77bbdc6220c57a06c3d267c0223/public/brca_metabric), which includes over 1900 patients annotated for tumor survival and tumor subtype (41). Each gene selected for the DC1 or DC2 signature showed a much stronger correlation with the indicated subtype than with the other subtype (Figure 1, A and B). Patients were divided into the top and bottom quartile for each signature, and surprisingly, we found that DC2 infiltration strongly correlated with improved survival, while there was no significant correlation with DC1 infiltration and only a weak correlation with the immune response (Figure 1C). This unexpected trend was also seen within the TCGA breast cancer data set, with the DC2 signature correlating more strongly with increased survival than the DC1 signature or the immune response score (Supplemental Figure 1A; supplemental material available online with this article; https://doi.org/10.1172/jci.insight.145885DS1). Our findings provide a different result than has previously been reported regarding the prognostic value of DC1 signatures, which have shown strong correlations with improved survival in melanoma (37, 38). We hypothesized that the prognostic value of DC subsets is influenced by the immune context of the tumor. ER^–^ tumors are reported to be “immune enabled,” while ER^+^ tumors have been termed “immune disabled” based on the prognostic value of the immunity signature (15). We found that DC2 infiltration specifically correlated with survival in the “immune disabled” ER^+^ tumors but not the ER^–^ tumors (Figure 1, D and E). DC1 infiltration, however, showed no effect in either subtype. We next sought to determine if DC2s influenced tumor aggressiveness. DC2 infiltration showed a significant inverse correlation with a proliferation signature (from ref. 15), while DC1 had no association (Figure 1F and Supplemental Figure 1B). Interestingly, we found that the proliferation signature was strongly correlated with poor outcomes in ER^+^ tumors but not ER^–^, mirroring the effect of DC2 infiltration within these 2 populations (Figure 1G). Our analysis reveals that DC2 plays a central role in the control of ER^+^ breast cancer.

**Figure 2:**
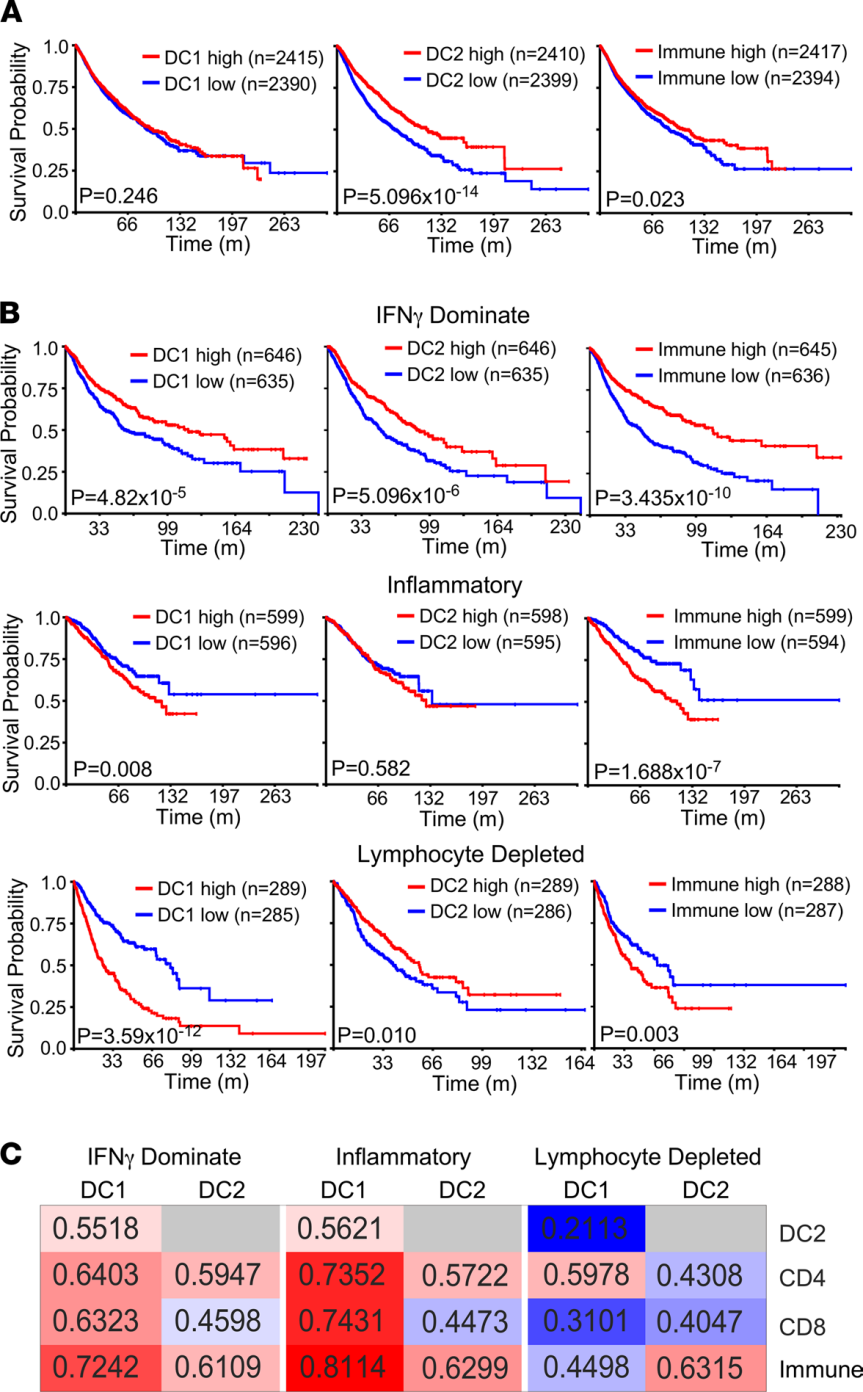
The benefit of DC subtypes within breast cancer is dependent on the immune context. (**A**) Kaplan-Meier curves for the overall survival of patients within the top or bottom quartile for the indicated gene signatures. In total, 10,593 primary tumors from the TCGA Pan-Cancer Atlas data set were used for analysis. (**B**) Kaplan-Meier curves for overall survival of patients within the top or bottom quartile for the indicated gene signatures from the TCGA Pan-Cancer Atlas. Tumors were segregated by the indicated immune subtype. (**C**) Heatmap reflecting the correlation of DC1 and DC2 gene signatures with the indicated immune populations. *R* values are listed in each cell. From the TCGA Pan-Cancer data set. (**A** and **B** were analyzed by a log-rank test).

Next, we sought to determine if our DC gene signatures could predict survival within specific immune contexts, regardless of tumor type. We analyzed patient data from the TCGA Pan-Cancer Atlas (http://xena.ucsc.edu) that consists of over 10,000 tumors representing over 33 forms of cancers. When all tumor types were batched together, DC2-high tumors (top quartile) showed significantly improved survival compared with DC2-low (bottom quartile), while the DC1 and immune response signatures displayed no effect on survival (Figure 2A). Six major immune environments have been identified within this data set (42). We sought to determine how lymphocyte infiltration affected the prognostic value of DC subtypes within the tumor by comparing T cell–rich (IFN-γ–dominant and inflammatory) and T cell–poor tumors (lymphocyte depleted; Figure 2B). DC signatures displayed a similar effect on survival within IFN-γ–dominant tumors, which are highly infiltrated by CD8^+^ T cells and M1 macrophages. However, within the Th1/17-rich inflammatory tumors, DC1-high and immune-high patients displayed a slight survival disadvantage. Surprisingly, within lymphocyte depleted tumors, DC1-high patients displayed drastically worse survival than DC1-low patients, while DC2-high showed slightly improved survival. Within inflammatory and IFN-γ–dominant tumors, DC1 infiltration strongly correlates with T cell infiltration and the immune response signature; however, the correlation of DC1 with CD8^+^ T cell infiltration was substantially lower in lymphocyte-poor tumors. (Figure 2C). Consistent with its effect on survival, the DC2 signature showed an equally strong correlation with CD4^+^ T cells and the immune response signature across all immune subtypes. These findings suggest that, within tumors with classically poor immune environments, DC1s are unable to generate a productive immune response, while DC2s are able to overcome the CD8-poor M2-high environment to stimulate immunity. Similar findings were also seen within the METABRIC data set. DC2 infiltration displayed a stronger correlation with survival within immune response–low patients (bottom tertile) compared with immune response–high patients (top tertile; Supplemental Figure 1, C and D). Our surprising findings reveal that the tumor immune contexture heavily influences the significance of DC subtypes within tumors.

### Defining DC populations in tumor and dLN.

Our human tumor data set analysis strongly suggests that DC2s are integral to the control of tumors that are resistant to CD8 infiltration. Therefore, we investigated the role of DC subtypes within the mouse mammary tumor virus-polyoma middle tumor antigen (MMTV-PyMT) breast cancer model, which yields aggressive tumors mirroring the properties of human triple-negative breast cancer (43). This model is nonresponsive to ICB therapy and has poor neoantigen expression (8, 17, 44). Immunocompromised mice with deficiencies in CD8^+^ T cell activity display similar growth rates for MMTV-PyMT tumors as WT mice, indicating that these tumors have completely escaped CD8 surveillance. We analyzed DC1 (MHCII^+^CD11c^+^Xcr1^+^) and DC2 (MHCII^+^CD11c^+^CD172^+^) subtypes within the primary (tumor) and secondary (draining lymph node [dLN]) sites of tumor immunity (gating scheme is shown in Supplemental Figure 2A) of tumor-bearing mice 3 weeks after implantation. The 2 DC subtypes displayed similar levels of maturation status (MHCII and CD86) at both sites; however, DC1s expressed inhibitory receptors (TIM3, BTLA, PD-L1) at a much higher level compared with DC2 (Supplemental Figure 2B). Interestingly, DC1s also expressed higher levels of CCR7, indicating that they have the potential for greater dLN-bound migration than DC2s. Indeed, in the tumor dLN, DC1s comprised a substantially higher proportion of the migratory population (15%) than in tumors (5%; Supplemental Figure 2C). These results indicate that DC1s migrate out of tumors with increased efficiency compared with DC2s.

### DC1 blocks antitumor immunity in breast cancer.

**Figure 3:**
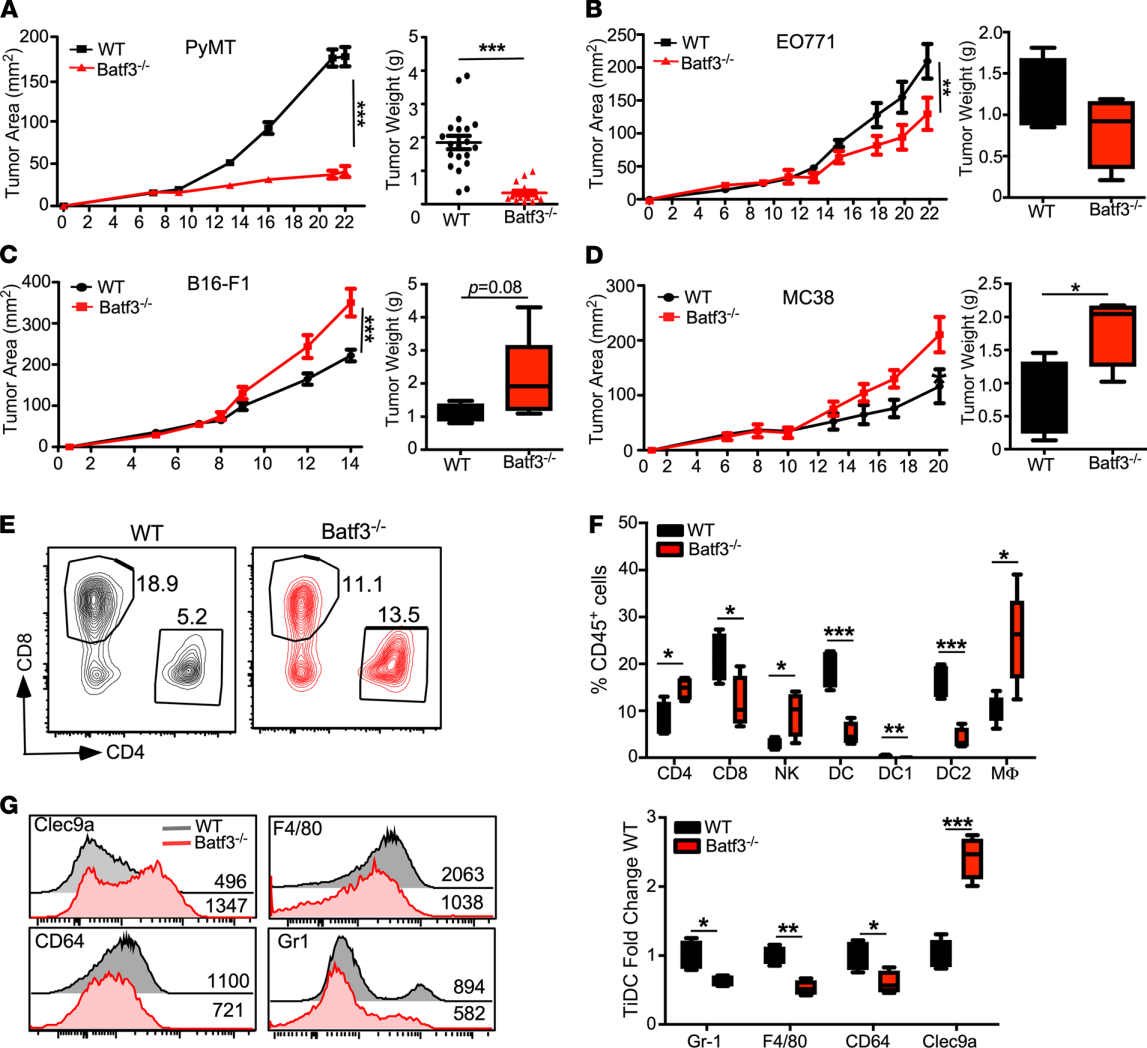
Loss of DC1 restores immune control of breast tumors. (**A**) Growth of PyMT breast tumors in C57BL/6 (WT) and Batf3^–/–^ mice. Tumor area (left) is from 1 representative experiment of 4 total. Tumor weight (right) is pooled results from all 4 experiments, WT (*n =* 20), and KO (*n =* 16). (**B**) Tumor growth of EO771 breast tumor implanted into fourth mammary gland in WT and Batf3^–/–^ mice. Tumor area (left) and tumor weight (right), *n =* 4 mice per group. (**C**) Tumor growth of B16-F1 melanoma implanted s.c. in WT and Batf3^–/–^ mice. Tumor area (left) and tumor weight (right), *n =* 5 to 6 mice per group. (**D**) Tumor growth of MC38 colon tumor implanted subcutaneously. Tumor area (left) and tumor weight (right), *n =* 5–6 mice per group. (**E**) Frequency of tumor-infiltrating T cells analyzed at end point. Representative plot displaying CD45^+^CD11b^–^NK1.1^–^CD3^+^ cells. (**F**) Frequency of tumor-infiltrating immune cell populations, analyzed at the end point. Data displayed are from 1 of 4 trials, *n =* 4. (**G**) Histograms of surface expression (MFI) of indicated receptors (left) on tiDCs (CD45^+^B220^–^CD11c^+^MHCII^+^). The bar graph (right) is the tiDC2 phenotype for expression of indicated markers from WT and Batf3^–/–^ mice. Data are represented as mean ± SEM. *P < 0.05, **P < 0.01, ***P < 0.001. (**A**, **B**, **C**, and **D** were analyzed by 2-way ANOVA for tumor growth; quantification was analyzed by 2-tailed unpaired *t* test).

High expression of inhibitory receptors on DC1 in breast tumors could negatively impact antitumor immune response (33). With increased DC1 migration toward dLN in the PyMT model, we hypothesize that this population could compromise antitumor immunity. To investigate the specific contribution of DC1 to the immune environment within PyMT tumors, we implanted PyMT tumor cells into the mammary glands of Batf3^–/–^ mice, which have significantly impaired DC1 development (ref. 45 and Supplemental Figure 3A). DC1 depletion dramatically attenuated tumor growth, with Batf3^–/–^ mice displaying an 80% reduction in tumor size at 3 weeks after implantation (Figure 3A). To determine if this finding is unique to the PyMT immune environment, Batf3^–/–^ mice were implanted with EO771 breast cancer cell line, and they displayed the same attenuated growth as PyMT cells (Figure 3B). Further, Batf3^–/–^ mice were implanted with cell lines that displayed higher levels of immunogenicity. Consistent with previous studies, both B16-F1 melanoma cells and MC38 colon cancer cells grew faster in Batf3^–/–^ mice (Figure 3, C and D). These results indicate that it is the intrinsic properties of breast tumors that affect DC subtype functions.

We next sought to determine the effect of DC1 depletion on the immune microenvironment of PyMT tumors. A comprehensive analysis of the immune microenvironment revealed an expected increase in the ratio of CD4/CD8 tumor-infiltrating T cells, with the frequency of CD4^+^ cells more than doubling in the Batf3^–/–^ mice (Figure 3E). Along with CD4^+^ T cells, there was increased infiltration of NK cells and TAMs (CD11c^–^CD11b^+^F4/80^+^; Figure 3F). Somewhat unexpectedly, we found that there was a significant reduction in tiDC2s. Furthermore, tiDC2s displayed reduced expression of receptors associated with monocyte lineage (F4/80, CD64, and Gr1) and increased expression of cDC marker Clec9a (Figure 3G), an antigen uptake receptor (46). Since monocyte-derived DCs are poor initiators of immune responses (47), the shift in the lineage of tiDC2s from monocytic to conventional suggests an improved ability to prime CD4^+^ T cells. We investigated DC2 phenotypes in the spleen of Batf3^–/–^ mice in order to determine if the changes seen within tiDC2s could be caused developmentally by the loss of Batf3. We found that the change in DC2 phenotype was not conserved outside the tumor, with only CD64 expression showing a decreasing trend while the other myeloid markers and Clec9a did not have any significant change (Supplemental Figure 3, B and C). These findings further suggest that improving tiDC2 functionality will enhance control of immune-poor tumors; however, they also surprisingly reveal that tiDC1 contributes significantly to the impaired immune response within these tumors.

### CD4^+^ T cells drive tumor immunity in MMTV-PyMT breast tumors.

**Figure 4:**
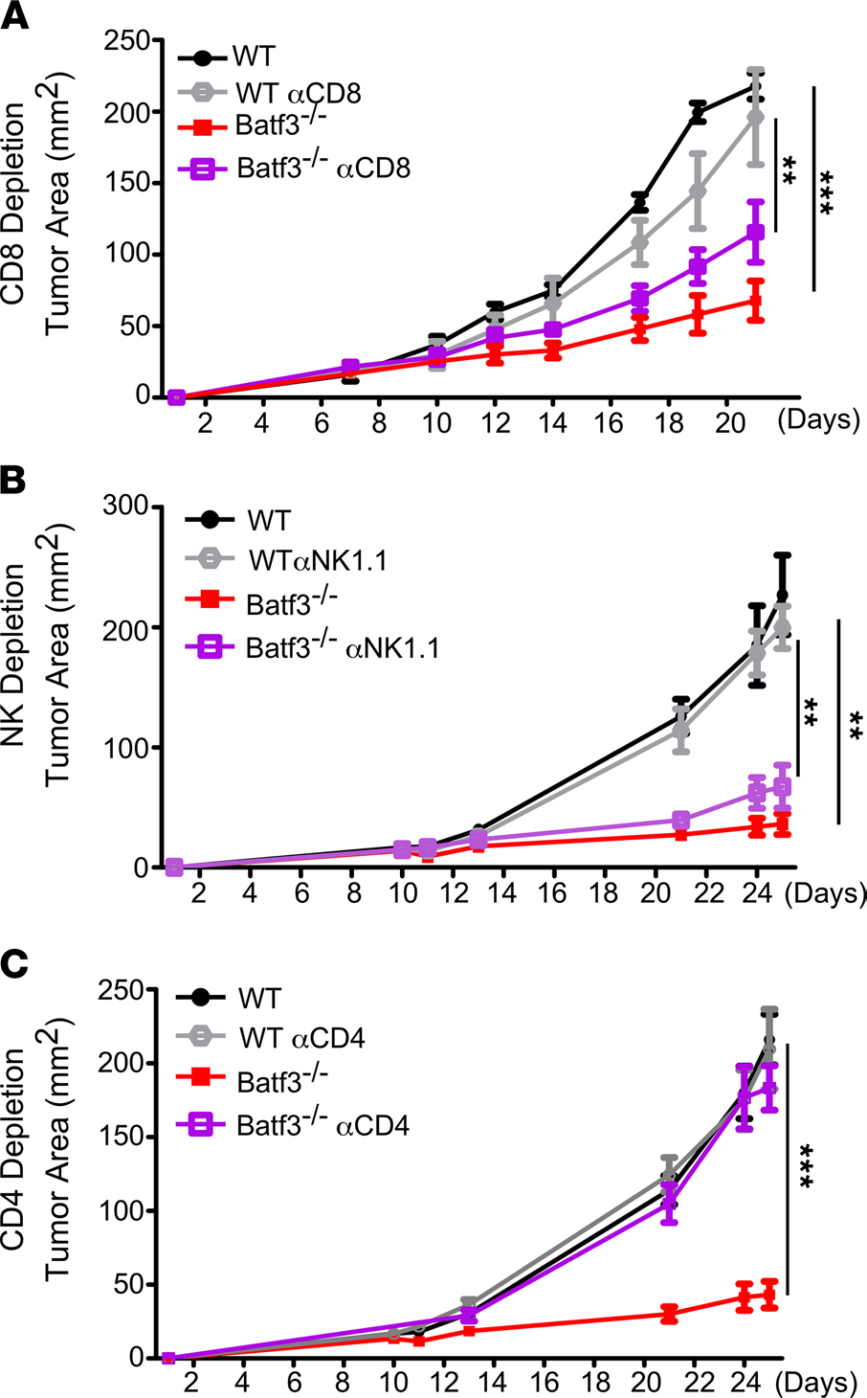
CD4^+^ T cells are essential for tumor immunity in Batf3^–/–^ mice. (**A**) PyMT tumor growth in WT or BATF3^–/–^ mice with or without CD8 depletion. *n =* 3–4 per group with 1 of 2 representative experiments shown. (**B**) PyMT tumor growth in WT or BATF3^–/–^ mice with or without NK cell depletion. *n =* 4–5 per group. (**C**) PyMT tumor growth in WT or BATF3^–/–^ mice with or without CD4 depletion. *n =* 3–4 per group with 1 of 2 representative experiments shown. Data are shown as mean ± SEM. **P < 0.01, ***P < 0.001 (2-way ANOVA).

The effector cells responsible for improved tumor control in Batf3^–/–^ mice were unclear and needed further investigation. Despite a general loss of cross-presentation in Batf3^–/–^ mice, cytotoxic T cells could still actively contribute to tumor surveillance through activation by nonclassical cross-presenting DCs and classical DC1s generated by Irf8 compensating for Batf3 deficiency (30, 48, 49). To address this possibility, mice were injected with CD8-depleting antibody prior to tumor cell implantation, but there was no effect on tumor growth in the Batf3^–/–^ mice, confirming that cytotoxic T cells are dispensable for control of PyMT tumors (Figure 4A). NK cells are also capable of directly killing tumors cells, and increased accumulation was seen in BATF3^–/–^ tumors (Figure 3E). To determine if they are playing a major role, NK cells were depleted prior to tumor cell implantation, but it did not impact PyMT tumor growth in either WT or Batf3^–/–^ mice (Figure 4B), indicating that NK cells are also dispensable for primary tumor growth. Having ruled out the cytotoxic effectors, we hypothesized that increased CD4^+^ T cell tumor infiltration is key to the antitumor response in Batf3^–/–^ mice. Indeed, when CD4^+^ T cells were depleted, immune control of PyMT tumors in Batf3^–/–^ mice was completely lost (Figure 4C), indicating that CD4^+^ T cells alone drive an effective antitumor response. Interestingly, depleting any of the effector populations did not affect tumor growth in WT mice, highlighting that the presence of DC1s in itself is sufficient to cause the complete immune escape of these tumors.

We next investigated the presence of lymphoid and myeloid suppressive cell populations that are reported to shape the microenvironment of breast tumors (16). The intratumoral and dLN CD4^+^ T cell compartment of Batf3^–/–^ to WT mice contained similar ratios of Foxp3^+^ Tregs (Supplemental Figure 4, A and B). Similarly, there was little difference in tumor infiltration of immature myeloid cells (CD11b^+^Ly6c^+^MHCII^lo^) in Batf3^–/–^ mice compared with WT mice (Supplemental Figure 4, C and D). Tumor infiltration of Ly6c^+^Ly6g^–^ monocytic myeloid-derived suppressor cells (MDSCs) was increased, whereas Ly6c^+^Ly6g^hi^ granulocytic cells remained unchanged (Supplemental Figure 4, C and D). Therefore, antitumor immunity in Batf3^–/–^ mice could not be attributed to the loss of any previously reported suppressive cell population.

### Migration of stimulatory DC2s leads to activation of antitumor myeloid cells.

**Figure 5:**
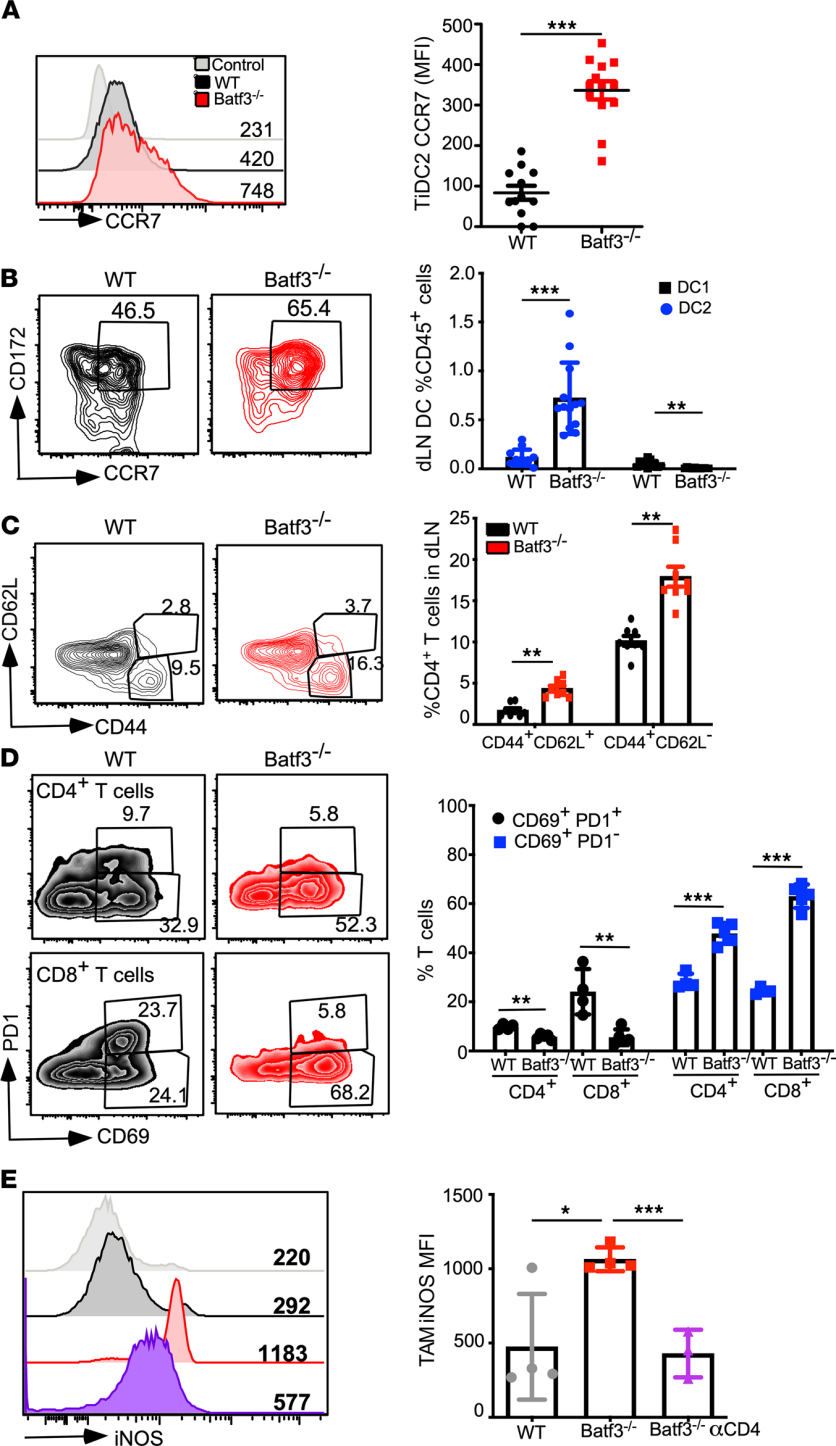
DC1 depletion improves DC2 migration and enhances T cell priming. (**A**) Representative histograms displaying CCR7 surface expression on tiDC2s at the end point (left). *n =* 12–13 per group, with 1 of 4 representative experiments shown (right). (**B**) Representative flow plot of dLN DCs (left) and quantification of the frequency of migratory DC populations (CD45^+^B220^–^MHCII^hi^CCR7^+^CD11c^+^ cells) (right). *n =* 12–13 per group, with 1 of 4 representative experiments shown. (**C**) Representative flow plot of CD4^+^ T cells from the dLN (left) and quantification of the frequency of different CD4^+^ T cells (right). *n =* 8 per group, with 1 of 4 representative experiments shown. (**D**) Representative flow plots of tumor-infiltrating CD4^+^ and CD8^+^ T cells for expression of PD-1 and CD69 (left), and the quantification of the data (right). *n =* 4–5 per group. (**E**) Representative histogram showing expression of iNOS in TAMs, MFI inset (left), and quantification of data (right). *n =* 3–4 per group, with 1 of 2 representative experiments shown. Data are shown as mean SEM. *P < 0.05, **P < 0.01, ***P < 0.001 (2-tailed unpaired *t* test).

The central role of CD4^+^ T cells for control of PyMT tumors in Batf3^–/–^ mice could be a bystander effect of an overall stimulatory tumor microenvironment or a de novo T cell response generated directly by tiDC2s. The changes of the cDC2 phenotype (Supplemental Figure 2B and Figure 3F) led us to predict that the tiDC2s in Batf3^–/–^ mice are better equipped to migrate to the dLN and prime CD4^+^ T cells. CCR7 expression on tiDC2s was increased by 3-fold (Figure 5A), and the number of CCR7^+^MHCII^hi^ migratory DC2s in the dLN was increased by 7-fold (Figure 5B) in Batf3^–/–^ mice. Increased migration of DC2s could account for the reduction in their population within the tumor (Figure 3E). Furthermore, both CCR7 expression on tiDC2s and expansion of the dLN migratory DC2s were not lost following CD4 depletion, demonstrating that DC2 migration is upstream of CD4^+^ T cell infiltration to the tumor (Supplemental Figure 5, A and B).

We next investigated if increased DC2 migration was a developmental side effect of Batf3 deficiency or if it was determined by the tumor microenvironment due to DC1 depletion. CCR7 expression was increased only on tiDC2s and not lymphoid-resident populations of the spleen or LN (Supplemental Figure 5C), ruling it out as a developmental defect. Furthermore, the deletion of Batf3 results in aberrant expression of DC1 transcription factor Irf8 within a subpopulation of DC2s. Irf8^hi^ DC2s were detected within the tumor and within the dLN resident DC population (CD11c^+^MHCII^int^CCR7^–/lo^; Supplemental Figure 5C). However, CCR7 expression is increased only in the Irf8^–/lo^ cells and not in the Irf8^hi^ population of DC2. Hence, Irf8 aberrant expression is not linked to CCR7 expression. Taken together, our findings indicate that the unique migratory phenotype of tiDC2s from Batf3^–/–^ mice is likely the result of tumor microenvironment changes and is not caused by altered DC development.

As a functional outcome of enhanced DC2 migration, CD4^+^ T cell activation was significantly augmented in the dLN and tumor of Batf3^–/–^ mice. The frequency of CD44^hi^ (Figure 5C) and the number of CD69^+^ICOS^+^Foxp3^–^ Th1-like CD4^+^ T cells doubled (Supplemental Figure 5, D and E). Importantly, PD-1 expression was significantly reduced on activated T cells (Figure 5D). The results indicate that the loss of DC1s leads to improved DC2 migration and function, which in turn better prime and sustain tumor-specific T cells.

By further analyzing the effector mechanism of antitumor responses, we found an increase in TAMs within the Batf3^–/–^ mice (Figure 3E). We examined whether these TAMs had been polarized to a proinflammatory M1-like phenotype, considering the augmented CD4^+^ T cell activation. The majority of TAMs (CD45^+^CD11b^+^CD64^+^ cells) from BATF3^–/–^ tumors expressed inflammatory marker iNOS compared with little to no iNOS detection in WT TAMs (Figure 5E). M1 polarization was completely dependent on CD4^+^ T cell activation, as CD4 depletion abrogated this effect (Figure 5E). Interestingly, the iNOS^+^ inflammatory TAMs were detected only in the tumor microenvironment (Supplemental Figure 6, A and B). In summary, these findings reveal a unique model for antitumor immunity identifying DC1 as the primary suppressive immune cell population within the breast cancer environment. CD4^+^ T cells are, surprisingly, much more effective at controlling tumor growth within this model than CD8^+^ T cells.

### Targeting DC-specific GP96 leads to a delay in the progression of spontaneous breast tumors.

**Figure 6:**
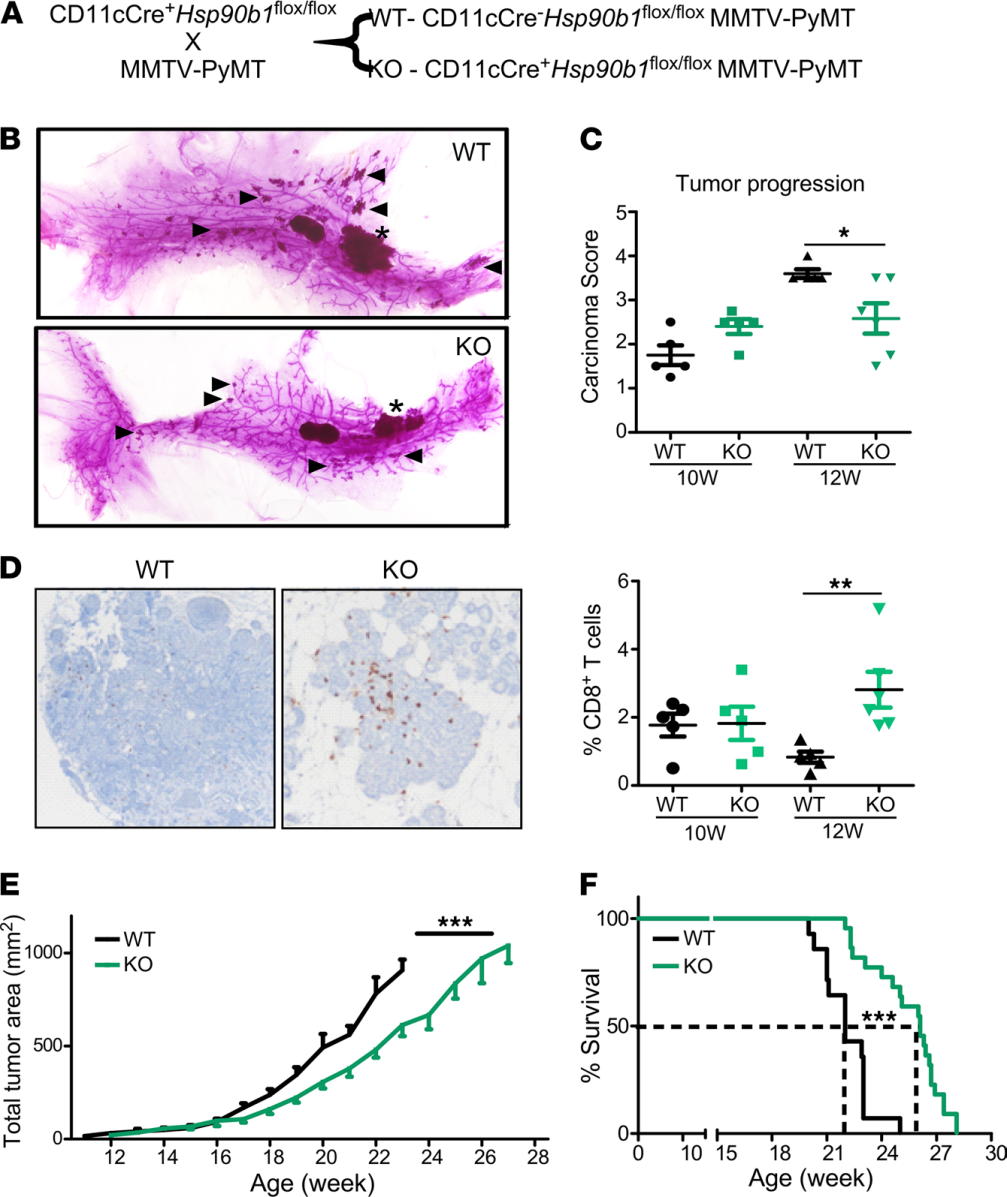
GP96 KODC delay progression of spontaneous breast tumors. (**A**) Diagram for the generation of DC-specific GP96 KO mice that develop spontaneous breast cancer. (**B**) Carmine stain of whole mounts of fourth mammary gland at 12 weeks of age. Asterisk marks primary carcinoma, and triangles mark select secondary sites. (**C**) Carcinoma score of H&E-stained mammary gland sections from 10- and 12-week-old mice. *n =* 4–8 per group. (**D**) IHC staining for CD8^+^ T cell infiltration into neoplastic regions from mammary glands of 12-week-old mice. Quantification of the percent of cells within tumor sites that are positive for CD8. *n =* 5–6 per group. (**E**) Total tumor area of WT and KO MMVT-PyMT mice: WT (*n =* 9) and KO (*n =* 14). (**F**) Survival graph showing age at which mice reach the humane end point: WT (*n =* 14) and KO (*n =* 22). Data are shown as mean ± SEM. *P < 0.05, **P < 0.01, ***P < 0.001. (**C** and **D** were analyzed by 2-tailed unpaired *t* test, **E** was performed using 2-way ANOVA, **F** was analyzed using a log-rank test).

The role of DC subtypes in shaping the immune landscape of breast tumors reveals potentially novel therapeutic targets for the development of immunotherapies. Previously, our lab identified a molecular target that selectively impacts the development and maturation of DC1s. Genetic deletion of *Hsp90b1* (encoding molecular chaperone GP96) from CD11c^+^ cells results in the loss of tolerogenic DC1s, an increase in inflammatory DC2s, and expansion of Tbet^+^CD4^+^ Th1 cells (50). Based on these findings, we sought to target GP96 within tumor DCs to test our hypothesis further that improving tiDC2 functionality will enhance the control of poorly immunogenic breast tumors. We crossed CD11cCre *Hsp90b1^fl/fl^* mice with MMTV-PyMT transgenic mice to determine the ability of GP96-null DCs (GP96 KODCs) to control spontaneous breast cancer (Figure 6A). To investigate tumor initiation, mice were sacrificed before the development of palpable tumors (10 or 12 weeks old). Analysis of mammary gland whole mounts revealed similar numbers of large and small tumor foci in WT versus KO (Figure 6B). Histologically, these foci correlated with variably sized regions of mammary intraepithelial neoplasia (MIN) and mammary carcinoma. A scoring system on a scale of 0–7 was used to compare carcinoma development across individual genotypes. Carcinoma scores were much larger in the WT mice by 12 weeks of age (Figure 6C), suggesting delayed tumor progression in KO mice. Therefore, tumor incidence is similar between WT and KO mice, but the progression is significantly slower in KO mice.

IHC analysis of the mammary glands revealed that GP96 KODCs improved local immune responses in the tumor. KO mice displayed a significant increase in T cell infiltration specifically into carcinoma regions at 12 weeks old, mirroring the difference in tumor growth (Figure 6D). These results suggest that KODCs are capable of initiating de novo immune responses in immunologically cold tumors. Furthermore, we found that tumors continued to grow substantially slower in KO mice, and they survived almost 4 weeks longer than WT control mice (Figure 6, E and F). These data reveal that targeting GP96 within tumor DCs leads to increased local immune activation and tumor control, and produces long-term survival benefits.

### Targeting DC-specific GP96 improves antitumor immunity.

**Figure 7:**
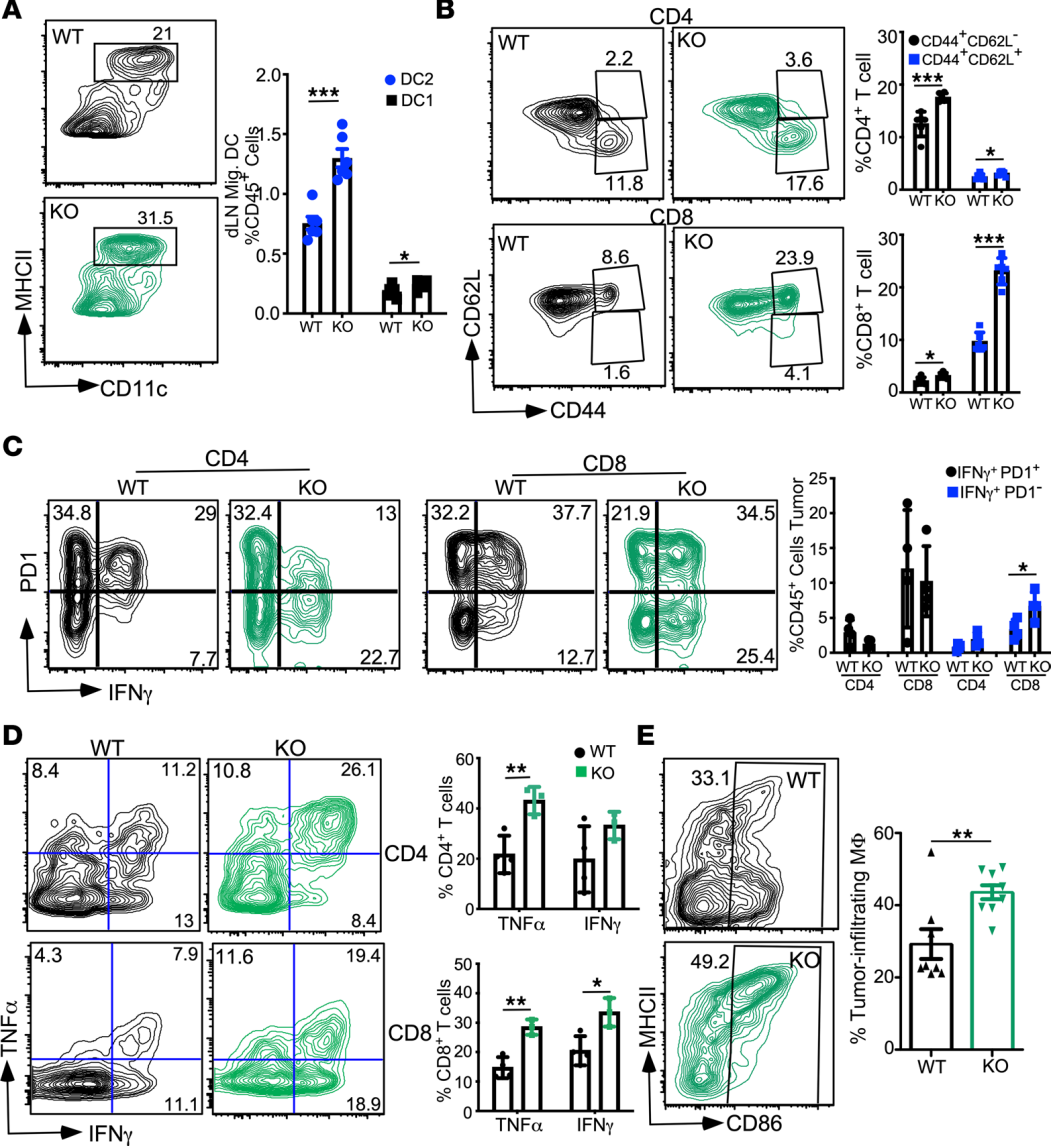
GP96 KODCs display enhanced T cell priming and maintenance. (**A**) Representative plot showing DCs from the draining LN of 18-week-old mice (left), and quantification of the frequency of migratory DC populations (CD45^+^B220^–^MHCII^hi^CCR7^+^CD11c^+^ cells) (right). *n =* 6 per group. (**B**) Representative plot showing CD44 and CD62L expression in CD4 (top) and CD8 (bottom) T cells from the dLN of 18-week-old mice, and quantification of data (left). *n =* 6 per group. (**C**) Representative plot showing PD-1 and IFN-γ expression on tumor-infiltrating CD4 (left) and CD8 (middle) T cells from 18-week-old mice, along with quantification of data (right). T cells were stimulated with PMA and ionomycin for 4 hours before staining. *n =* 4 per group. (**D**) Representative flow plots of TNF-α– and IFN-γ–producing cells in tumor-infiltrating CD4 (top) and CD8 (bottom) T cells at the end point (24- to 28-week-old mice) (left). Quantification of data (right). T cells were stimulated with PMA and ionomycin for 4 hours before staining. *n =* 3–4 mice per group. (**E**) Representative plot showing tumor-infiltrating macrophages expression of CD86 and MHCII (left), and quantification of data (right). Data shows graph of M1-like polarization (CD86^+^). *n =* 8 to 9 mice/group. Data are shown as mean ± SEM. *P < 0.05, **P < 0.01, ***P < 0.001 (2-tailed unpaired *t* test).

Next, we determined if the T cell response induced by KODC is maintained at the later stage of PyMT tumors. Tumor-bearing mice were sacrificed at 18–20 weeks old, and dLN and tumors were immunophenotyped (at this time point, average tumor size was 176 mg for WT and 120 mg for KO). GP96-depleted DCs were identified by the loss of its client proteins CD11c and CD11b. We found that DC infiltration was similar in WT and KO mice (Supplemental Figure 7A). Similar to the Batf3^–/–^ model, GP96 KODCs (B220^–^MHCII^+^CD11c^–^CD11b^–^ cells) contain significantly fewer DC1s (Supplemental Figure 7B) and increased migrating DC2s in the dLN (Figure 7A). Consequently, T cell activation was enhanced within the dLN of KO mice (Figure 7B). Improved T cell stimulation was also seen in the tumor microenvironment in KO mice. Within WT mice, IFN-γ^+^ CD4^+^ and CD8^+^ T cells expressed high levels of PD-1, indicating the inability of antigen-presenting cells (APCs) to effectively maintain the T cell effectors. However, expression of PD-1 was significantly reduced in IFN-γ^+^ CD4^+^ and CD8^+^ T cells in KO tumors (Figure 7C).

At the endpoint (24–28 weeks old), even when tumors were large and necrotic, we found that both CD4^+^ and CD8^+^ tumor-infiltrating T cells still displayed an improved functional phenotype in KO tumors. Tumor-infiltrating T cells from KO mice produced significantly more IFN-γ and TNF-α (Figure 7D). Additionally, increased T cell expression of Ly6A/E (also known as Sca-1) in KO mice reflects an improved cytokine environment within KO tumors (Supplemental Figure 7, C and D). Along with the increased inflammatory cytokine production, there was an increase in M1-like polarization of TAMs (CD45^+^CD11c^–^F4/80^+^MHCII^hi^) within the KO tumors (Figure 7E). These findings identify GP96 as a potentially novel target for enhancing the therapeutic function of DCs.

## Discussion

DCs consist of distinct subsets specialized for stimulating unique aspects of the immune response. Gene signature analysis of DC subtype infiltration into human tumors within 2 large cancer data sets revealed that the immune contexture of tumors heavily influences the prognostic value of DC subtypes. Within T cell–resistant tumors (tumors with low CD8 infiltration and high M2-like macrophage signatures), DC2 infiltration displayed a substantially stronger correlation with survival than cDC1. Our findings were further supported by mechanistic studies in mice. Two unique mouse models (Batf3^–/–^ and the potentially novel CD11cCre *Hsp90b1^fl/fl^*) with varying levels of DC1 impairment enhanced the DC2–CD4^+^ T cell axis and improved control of highly suppressive PyMT tumors. In comparison with DC2, DC1 uptake antigen and migrate out of the tumor with increased efficiency in accordance with their higher expression of CCR7 and Clec9a. Upon deleting DC1 from the tumor microenvironment, DC2s were able to stimulate an effective CD4^+^ T cell–driven antitumor immune response, restoring spontaneous antitumor immunity. At 3 weeks after implantation, tumors in Batf3^–/–^ mice were roughly 20% of the size of tumors in WT mice. DC2s displayed decreased monocyte lineage markers and increased expression of cDC receptors, including CCR7 and Clec9a, leading to increased DC2 migration out of the tumor microenvironment. In addition, we found that CD4^+^ T cells were able to induce inflammatory activation of myeloid cells to substantially impair tumor growth. The loss of any of these compartments results in immune escape of the tumor and uncontrolled growth. Our findings highlight the power of the DC2–CD4^+^ T cell axis to control poorly antigenic tumors resistant to cytotoxic CD8^+^ T cells.

In contrast with our findings, numerous studies have found that DC1s are essential for the development of antitumor immunity. Broz et al. identified a BATF3-dependent DC1 population within numerous tumor models and found that they were important for CTL functions and adoptive T cell therapy (31). Follow-up studies using antigen-rich melanoma models revealed that DC1s control all aspects of the CTL response from antigen trafficking and CTL priming in the dLN to recruitment into the TME (30, 32); therefore, DC1s were unsurprisingly found to be essential for immune checkpoint therapy (ICT) and adoptive T cell therapy in these models. Interestingly, a recent study showed that cDC1s are also required for earlier CD4^+^ T cell priming, and CD40 signaling in cDC1 is critical for CD8^+^ T cell priming and CD4^+^ T cell activation against fibrosarcoma and melanoma (51). The differences between these studies and our results are likely due to the different tumor immune environments in the models studied. Melanoma tumors often have high mutation rates and are neoantigen-rich, making them good targets for CTL immune control (10). Unlike melanoma, PyMT breast tumors are poorly antigenic and display a total loss of spontaneous immune control, evidenced by tumors growing similarly in the absence of effector cell populations (Figure 4) (44). Further evidence of the difference between these environments can be seen in the role of NK cells. In melanoma models, studies showed that NK cells were essential for antitumor immunity through the recruitment of DC1s (37, 38). However, we found that NK cell depletion did not affect DC1 infiltration or the growth of PyMT breast tumors (Supplemental Figure 5 and Figure 4B). In contrast to these tumor studies, the suppressive properties of DC1s are well characterized in other disease settings (25), as they are potent inducers of Tregs in the antigen-rich environment of the gut. Our analysis of the Pan-Cancer Atlas further shows that the role of DC subtypes within tumors is immune context dependent. Infiltration of DC1 indicates improved outcomes within IFN-γ–dominant tumors but significantly worse outcomes in lymphocyte depleted tumors.

Most immunotherapies have solely focused on activating or enhancing CD8^+^ T cells for the induction of antitumor immunity. Their cytotoxic properties make them ideal targets (52, 53); however, several studies have found evidence that CD4^+^ T helper cells initiate and sustain antitumor immunity. In humans, Hodgkin’s lymphoma (HL) has among the highest response rates to ICB therapy (greater than 70%), but HL cells frequently downregulate MHCI expression, which indicates that CD4^+^ T cells may play an important role in generating tumor immunity in these patients (54). Mechanistic studies in mice have revealed that CD4^+^ T cells are key regulators of the functions of tumor-infiltrating myeloid cells. CD4^+^ T cells were found to induce macrophage-mediated tumor rejection in myeloma (55). In breast cancer, Th2-like CD4^+^ T cells protected tumors from chemotherapy and induced metastasis by inducing M2-like TAM polarization (56). Blocking macrophage infiltration or inducing a Th1 phenotype in CD4^+^ T cells significantly attenuates breast tumor growth. The deletion of Tregs from PyMT tumors enables a CD4^+^ T cell–dependent increase in inflammatory M1-like macrophage infiltration into the tumor-inducing spontaneous tumor immunity (16). Therefore, tumors rich in myeloid cells, which are particularly hostile to CD8 responses, make good candidates for CD4/DC2-targeted therapies. This finding is supported further by a study in mice from Laoui et al., who found that DC2 vaccination was more effective against TAM-rich LLC tumors than DC1 vaccination (47).

Immunological chaperone GP96 has been reported to regulate cells from both the myeloid and lymphoid lineages through its client protein network, which includes members of the TGF-β1, TLR, integrin, and Wnt signaling pathways (57–63). GP96 deletion, specifically from CD11c^+^ DCs, results in a DC population with increased stimulatory potential (50). This finding was unique to DCs, as deletion of GP96 from macrophages resulted in the decreased inflammatory potential of these cells (64). Genetic targeting of GP96 within PyMT breast tumors partially restored spontaneous immune control within these mice. Unlike WT controls, GP96 KODCs initiated the immune response earlier than WT controls, resulting in delayed outgrowth, increased DC2 migration, and T cell activation. In contrast to results seen in the Batf3^–/–^ model, CD11cCre *Hsp90b1^fl/fl^* mice displayed increased activation of both CD4^+^ and CD8^+^ T cells. This difference could be due to a larger population of cDC1s existing within the GP96 KO mice compared with Batf3^–/–^ mice, coupled with the improved immune environment generated by the enhanced DC2–CD4^+^ T cell axis. It’s also possible that GP96-depleted cDC2s directly prime CD8^+^ T cells. The mechanism by which GP96 regulates DC function is under current investigation. These findings show the ability to generate a local immune response to tumors through the systemic targeting of a pathway within DCs, highlighting GP96 as a potentially novel target for immunotherapy development. Targeting GP96 within tumor-infiltrating DCs appears challenging, but there are several strategies already under development, including small molecular inhibitors and small interfering RNA delivery (65–67). Furthermore, targeting GP96 in DCs ex vivo could lead to the development of improved DC vaccines.

Our work identified the contributions DC subsets make toward different tumor microenvironments. DC1s are critical for the development of antitumor CTL responses; however, in tumors that have microenvironments hostile to CD8^+^ T cells, DC1s can hamper the antitumor immunity. By interfering with DC2’s ability to uptake available antigens and respond to migratory stimuli, DC1 can become more of a hindrance under certain conditions. Targeting DC2s within these environments leads to improved DC migration, CD4^+^ T cell stimulation, and initiation of spontaneous tumor immunity. Our findings provide a rational basis for the development of DC subtype–targeted therapies and highlight the importance of characterizing the entire immune landscape of tumors for the comprehensive design of immunotherapy combinations.

## Methods

### Mice.

CD11c^+^ cell–specific GP96-deficient mice (CD11cCre^+^*Hsp90b1^fl/fl^*) and control littermates (CD11cCre^–^*Hsp90b1^fl/fl^*) were generated by crossing our *Hsp90b1^fl/fl^* (GP96 is encoded by *Hsp90b1*) mice (58) with CD11c-Cre transgenic mice (68). These GP96-deficient mice were further crossed with MMVT-PyMT transgenic mice (69) to generate MMVT-PyM-CD11cCre^+^*Hsp90b1^fl/fl^* mice. Batf3^–/–^ mice were purchased from The Jackson Laboratory (stock no. 013755). All mouse models in this study are on the C57BL/6 background.

### Reagents, cell lines, and software.

Antibodies used for flow cytometry were obtained from BD Biosciences, eBioscience, BioLegend, TONBO biosciences, and Thermo Fisher Scientific. Antibodies for immunohistochemical staining were purchased from Abcam and Cell Signaling Technology. Percoll was obtained from GE Healthcare Life Sciences. Dispase was purchased from Worthington. All other chemicals and culture medium were obtained from Sigma-Aldrich and Thermo Fisher Scientific (all information regarding antibodies is included in Supplemental Table 1). The PyMT breast cancer cell line was established by Beichu Guo at the MUSC. EO771 breast cancer cell line was provided by Stephen Tomlinson at MUSC. B16-F1 melanoma and MC 38 were obtained from ATCC and Kerafast. The information for software used in this study, including Flowjo (purchased from Flowjo LLC), GraphPad Prism (purchased from GraphPad), and Xena platform (http://xena.ucsc.edu/), is included in Supplemental Table 1.

### Analysis of human patient data.

Human patient data were analyzed using the publically available UCSC Xena Platform (70). The TCGA breast cancer and TCGA Pan-Cancer cohorts are available through the Xena Platform. We downloaded the METABRIC data set from cBioPortal’s public repository (71) on January 3, 2020 (https://github.com/cBioPortal/datahub/tree/ab7cb6294676e77bbdc6220c57a06c3d267c0223/public/brca_metabric), and analyzed the data through a local Xena hub. Overall survival analysis was performed using the top and bottom quartile of expression values of gene signatures. Significance was determined using a log-rank test. Correlation analysis was performed using Pearson’s correlation. Gene signatures that were used include the following: DC1 (XCR1, CLEC9A, BATF3), DC2 (FCER1A, CLEC10A, CD1C), proliferation (AURKA, BIRC5, CCNB1, CCNE1, CDC20, CDC6, CENPF, CEP55, EXO1, MKI67, KIF2C, MELK, MYBL2, NDC80, ORC6, PTTG1, RRM2, TYMS, and UBE2C), and immune (APOBEC3G, CCL5, CCR2, CD2, CD27, CD3D, CD52, CORO1A, CXCL9, GZMA, GZMK, HLA-DMA, IL2RG, LCK, PRKCB, PTPRC, and SH2D1A).

### Flow cytometry.

For flow cytometry analysis of tumor-infiltrating cells (TILs), tumor tissue was digested with a cocktail of collagenase D (2 mg/mL, Roche), DNase I (0.1 mg/mL, Sigma-Aldrich), and Dispase (0.075 μg/mL, Worthington) for 30 minutes, and then leukocytes were isolated using a 40%/80% Percoll gradient (GE Healthcare). The cells were isolated from the spleen and lymph node by mechanical disassociation. For staining surface receptors, cells were stained with viable dye, followed by FcR blocking for 15 minutes, and then with a cocktail of surface antibodies for 30 minutes. Cells were then fixed with 4% PFA. For intracellular staining of transcription factors and iNOS, after surface staining, the cells were fixed and permeablized using eBioscience Fix/perm kit according to the manufacturer’s instructions. For cytokine staining, cells were treated with Brefeldin A (5 μg/mL) and PMA (50 ng/mL)/ionomycin (1 μg/mL) for 4 hours before staining. Data were collected with an LSR Fortessa flow cytometer (BD Biosciences) and analyzed using FlowJo version 9 (FlowJo LLC).

### Tumor studies.

PyMT cells derived from a spontaneous mammary tumor from an MMTV-PyMT mouse were gifted from Beicho Guo (Medical University of South Carolina, Charleston, South Carolina, USA). For breast tumor growth studies, 4 × 10^5^ PyMT cells were injected directly into each of the fourth pair of mammary glands through a small incision in the skin in 20 μL PBS. Tumor area (length × width) was measured every 3–4 days, and the average tumor area was reported. For s.c. tumor studies, tumor cells (5 × 10^5^ for B16-F1 and 1 × 10^6^ for PyMT) were inoculated into the rear lower flank in 100 μL PBS. For T cell–depletion studies, 200 μg of αCD8 (clone 53-6.7, catalog BE0004-1, BioXCell) or αCD4 (clone GK1.5, catalog BE0003-1, BioXCell) antibody was administered i.p. on day –1, and then 100 μg was injected every 3 days until the end point. For the NK cell–depletion experiment, 200μg of αNK1.1 antibody (clone PK136, catalog BE0036, BioXCell) was administered i.p. on day –1, and then continue administrated once a week the experiment ended. For analysis of spontaneous tumor development from the MMTV-PyMT mice, we monitored mice for palpable tumors once a week at 10 weeks old and twice a week starting at 12 weeks old. Once the tumors were detected, the tumor area was measured twice per week until mice reached the humane end point.

### Histology.

The second and fourth pair of mammary glands were collected from 10- or 12-week-old MMTV-PyMT mice for histological analysis. The glands were fixed with 10% neutral buffered formalin (Sigma-Aldrich, catalog F1635) for 24–72 hours and then placed in 70% ethanol (Thermo Fisher Scientific, catalog NC-1000-1GL). Tissue sections were routinely processed and embedded for histology. Sections (4 μm thick) were placed on positively charged slides for H&E and IHC staining. H&E sections were reviewed by a veterinary pathologist, and a carcinoma score was obtained as follows: 0, no carcinoma present; 1, diameter of largest carcinoma ≤ one 400× field; 2, > one 400× field but ≤ one 200× field; 3, > one 200× field but ≤ one 100× field; 4, > one 100× field but ≤ one 40× field; 5, > one 40× field but ≤ one 20× field; 6, > one 20× field (add 1 if multiple foci of carcinoma are present in one gland). MINs were characterized by increased numbers of ductal structures that were well circumscribed from the adjacent stroma and lined by multiple layers of nonpolarized, cuboidal epithelium that often completely filled the ductal lumen. Carcinomas were characterized by polygonal epithelial cells arranged in poorly circumscribed, small nests or individual cells that were embedded within a reactive fibrotic stroma. IHC for CD8 (Cell Signaling Technology, catalog 98941) was performed on a Discovery Ultra (Roche; Ventana) autostainer using EDTA-based antigen retrieval, primary antibody incubation at a concentration of 1:100 at room temperature for 30 minutes, followed by Omni-prep anti–rabbit HRP secondary (Roche, catalog 760-4311) and DAB detection (Thermo Fisher Scientific, catalog NC1689706) and hematoxylin counterstain (Thermo Fisher Scientific, catalog TA-125-MH). Slides were scanned at 20× resolution with the Vectra Polaris (Akoya Biosciences) multispectral imaging system. Up to three 2 × 2 or 3 × 3 fields with the highest frequency of CD8^+^ T cells were manually selected and acquired for image analysis from each slide. Image analysis was performed through Inform software (Akoya Biosciences) to quantify the percentage of CD8^+^ T cells. MINs and carcinomas were analyzed separately. The mean percent of CD8^+^ T cells were reported for each mouse and compared across genotypes using a 2-tailed Student’s *t* test with α = 0.05.

### Whole-mount preparation.

Mammary gland whole mounts were prepared as previously described (72). Briefly, the second and fourth mammary glands were collected and spread out on a glass slide. They were fixed with Carnoy’s fixative overnight, washed, and then stained with Carmine Alum. The glands were imaged at 0.5× magnification with a Nikon SMZ Stereo Microscope using a Nikon DS-Fi3 camera. Quantification of tumor area was performed using ImageJ software (NIH).

### Statistics.

Statistical analysis was performed using Prism 8 (GraphPad). All data points represent biological replicates and are presented as the mean ± SEM. Growth curves were analyzed using a 2-way ANOVA. Pairwise comparisons were performed using a 2-tailed unpaired Student’s *t* test. Significance of survival results was determined using a log-rank (Mantel-Cox) test.

### Study approval.

All animal experimental protocols were approved by the MUSC and the Ohio State University (OSU) IACUC. All methods were carried out in accordance with federal regulation, as well as established institutional guidelines and regulations. Animals were maintained in the MUSC’s animal resource facility and OSU’s University Laboratory Animal Resources. All studies were approved by MUSC’s and OSU’s IACUCs.

## Author contributions

Conceptualization was contributed by SI and BL. Methodology was contributed by SI and BL. Formal analysis was contributed by SI, SN, CK, and BL. Investigation was contributed by SI, SN, YL, MH, CK, MP, and BL. Resources were contributed by BG and ZL. Writing of the original draft was contributed by SI and BL. Review and editing were contributed by SI, SN, MH, CK, BG, ZL, and BL. Supervision was contributed by BL. Funding acquisition was contributed by BL.

## Supplementary Material

Supplemental data

## Version 1 07/20/2021

In-Press Preview

## Version 2 09/08/2021

Electronic publication
