# Supplementary material for: Type 2 dendritic cells mediate control of cytotoxic T cell resistant tumors
Source: JCI Insight. 2021 Sep 8;6(17):e145885. doi: 10.1172/jci.insight.145885 (PMC8492342; doi:10.1172/jci.insight.145885)
Supplement: Supplemental data [file jciinsight-6-145885-s197.pdf]

SUPPLEMENTAL FIGURES
Figure S1

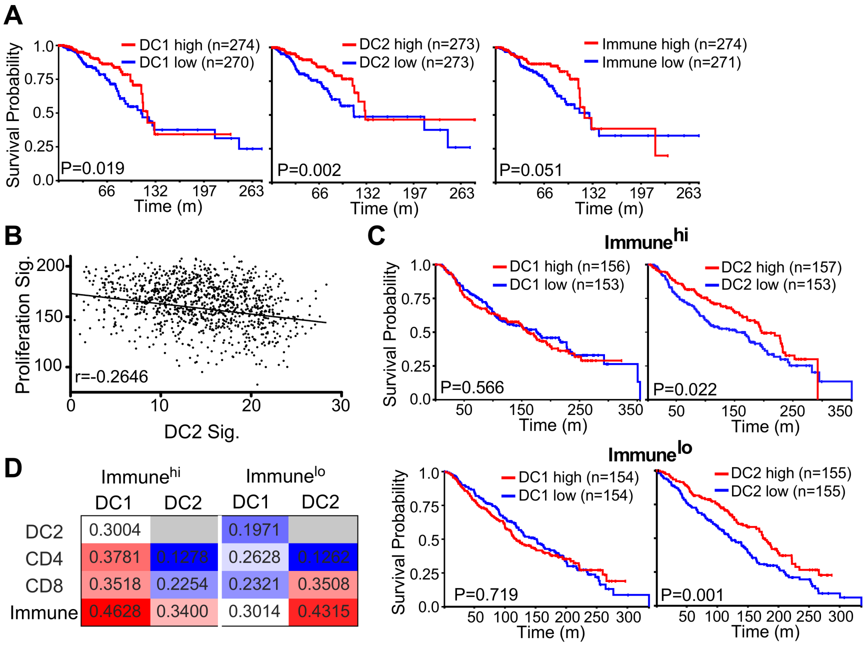

Figure S1. The benefit of DC subtypes within breast cancer is dependent on the immune context. (A) Kaplan Meyer curves for the overall survival of patients with breast cancers within the top or bottom quartile for the indicated gene signatures. 1101 primary breast tumors from the TCGA dataset were used for analysis. **(B)** Correlation of the proliferation gene signature with the signature for DC2s from the TCGA dataset. **(C)** Kaplan Meyer curves for the overall survival for the top or bottom quartile of the indicated gene signatures for breast cancer patients from the METABRIC dataset. Patients are divided into the top or bottom quartile for the immune response signature. **(D)** Heat map reflecting correlation of DC1 and DC2 gene signatures with the indicated immune populations. R values are listed in each cell. From METABRIC dataset. (A and C were analyzed by a log-rank test, B was analyzed using Persons correlation).

Figure S2

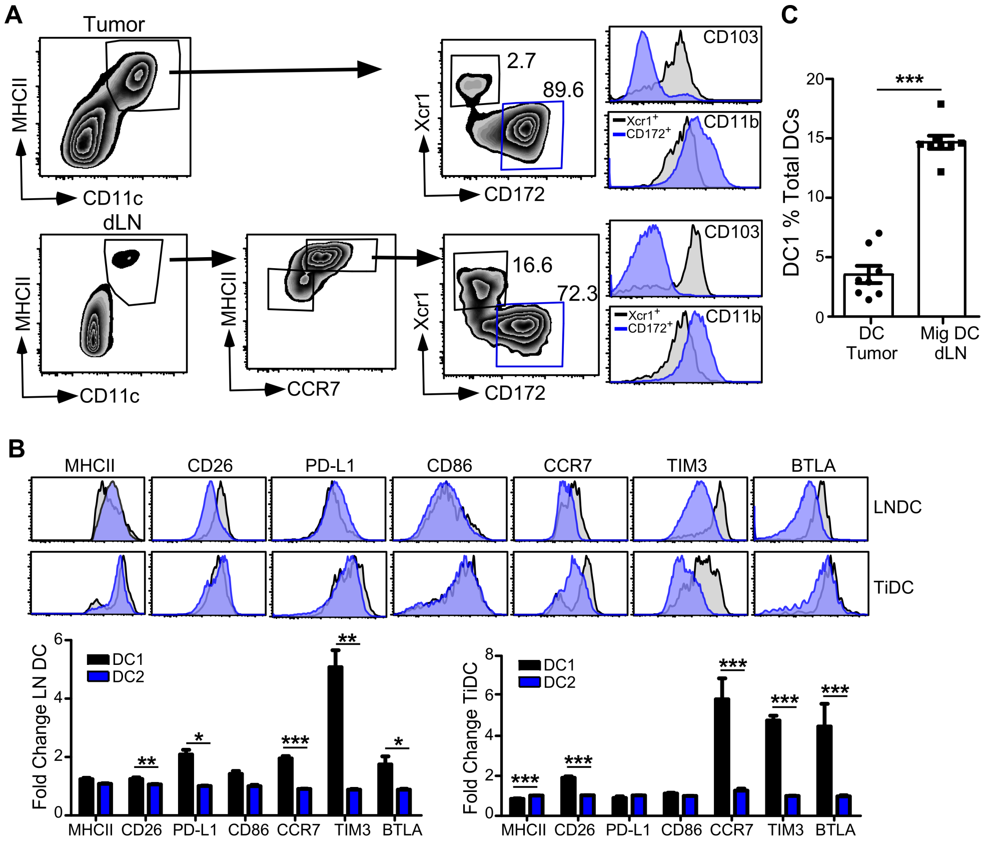

Figure S2. Defining DC populations in tumor and draining lymph node. (A) Flow ~~dating~~ scheme for tiDC (top) and migratory dLN (bottom) DC subtypes. **(B)** Top: Representative histograms of surface markers on total DCs (CD45^{+}B220^{-}CD11c^{+}MHCII^{+}). Bottom: Quantification of expression levels of indicated markers on DC1 (Xcr1^{+}) and DC2 (CD172^{+}) relative to their expression on total DCs. Results shown are from one of three experiments, n=12. **(C)** DC1 frequencies from TIL and dLN migratory DC compartments in PyMT tumor-bearing mice at the endpoint. n=8 per group one of four representative experiments shown. Data are shown as mean ± SEM. *p<0.05, **p<0.01, ***p<0.001. (2-way unpaired t-test).

Figure S3

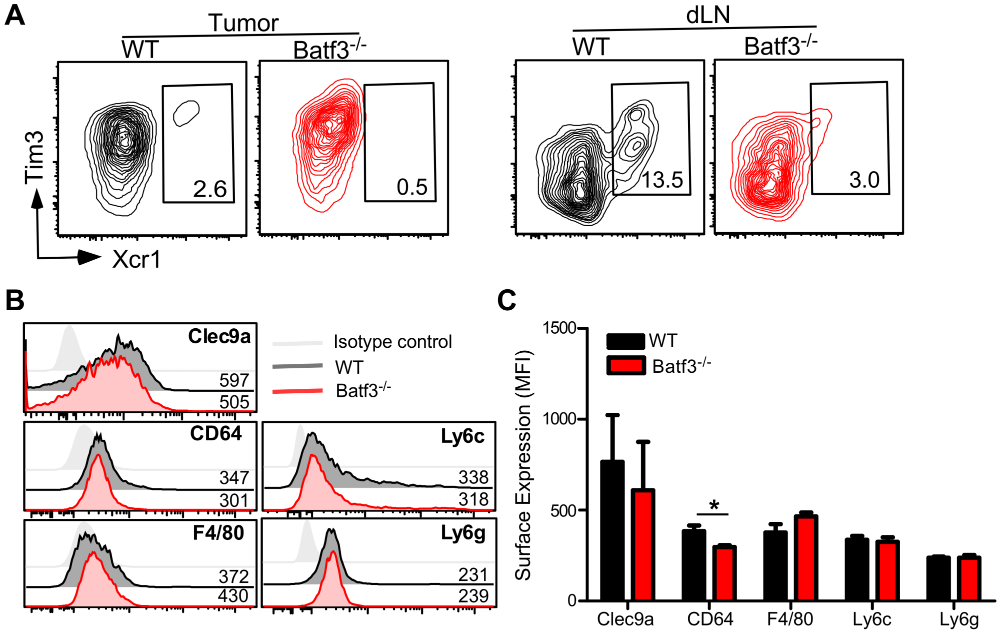

Figure S3. Loss of DC1 induces tumor-specific changes to DCs. (A) Representative flow plot of the frequency of DC1s in PyMT tumor-bearing WT and Batf3$^{-/-}$ mice. **(B)** Representative histograms displaying expression of surface receptors on Splenic DCs, MFI inset. **(C)** Quantification of (b), n=4 mice per group. Data are represented as mean ± SEM. * p<0.05. (2-way unpaired t-test).

Figure S4

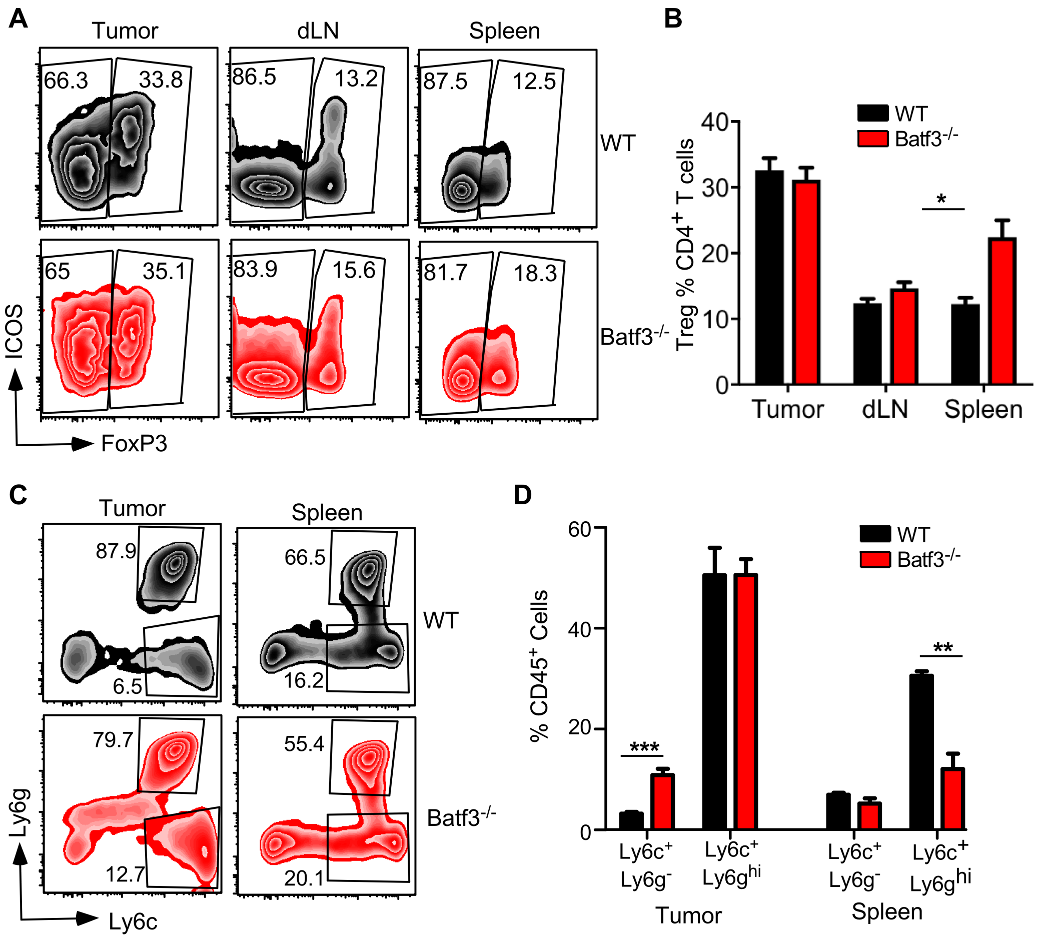

Figure S4. Batf3 KO does not reduce frequency of suppressive immune cell populations. (A) Representative flow plot of Foxp3$^+$ CD4$^+$ regulatory T cells within different tissues of PyMT tumor-bearing mice at the endpoint. n= 4-8 mice per group, one of four representative experiments shown. **(B)** Quantification of the frequency of Foxp3$^+$ CD4$^+$ regulatory T cells within different tissues of PyMT tumor-bearing mice at the endpoint. **(C)** Representative flow plot of immature myeloid cells (CD45$^+$ MHCII$^{Lo/-}$ CD11b$^+$) in tumor and spleen of PyMT tumor-bearing mice. n=4-5 Mice /group. **(D)** Quantification of the frequency of (c). Data are shown as mean ± SEM. *p<0.05, **p<0.01, ***p<0.001. (2-way unpaired t-test).

Figure S5

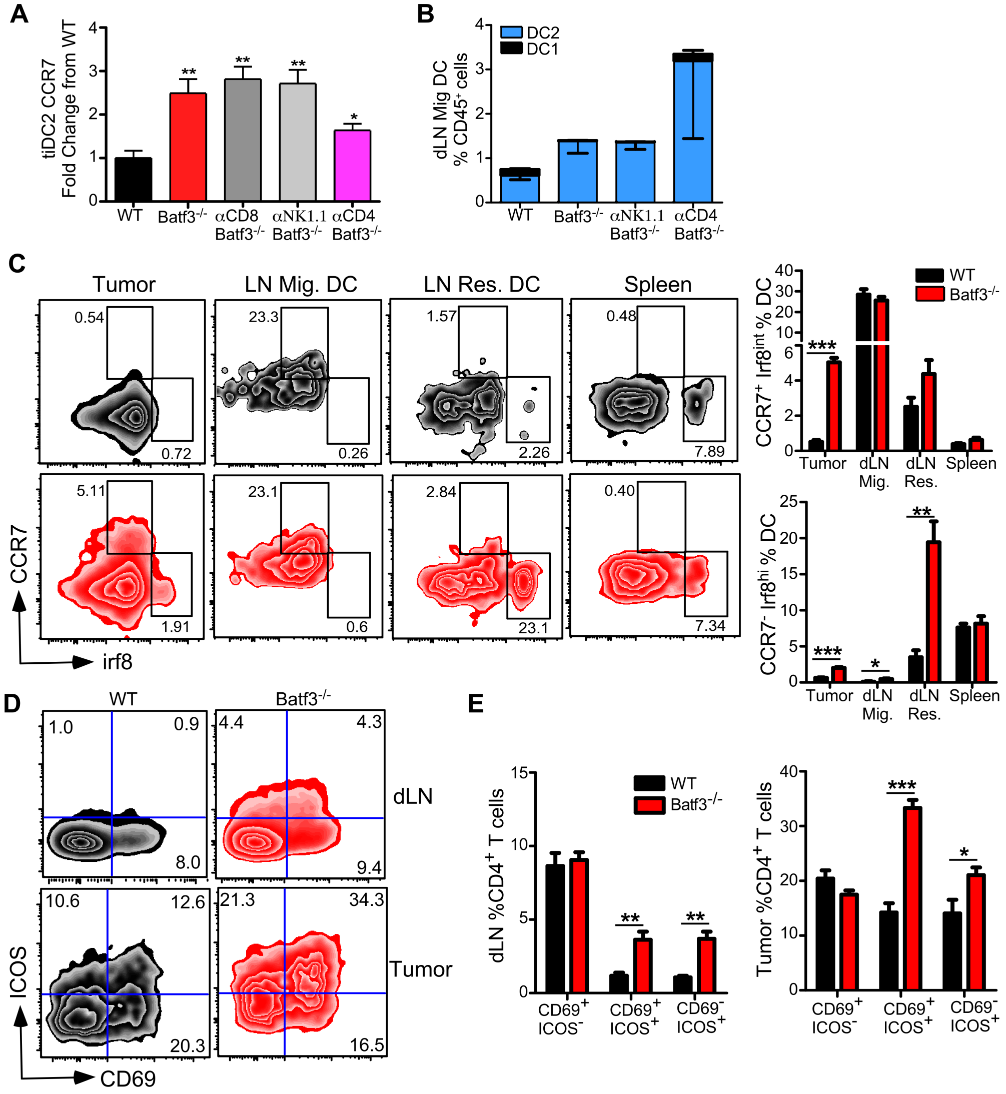

Figure S5. DC1 depletion improves DC2 migration and enhanced T cell priming. (A) CCR7 surface expression on tiDC2 at the endpoint, MFI from each group normalized to WT. n=3-5 per group. **(B)** Frequency of migratory DC populations in the dLN of PyMT tumor-bearing mice. n=3-8 per group. **(C)** Representative plots of Irf8 and CCR7 expression on DCs from tumor, spleen, dLN migratory DCs ($CD45^+$ $B220^-$ $CD11c^+$ $MHCII^{hi}$), and dLN resident DCs ($CD45^+$ $B220^-$ $CD11c^+$ $MHCII^{int}$). Quantification of $CCR7^+Irf8^{int}$ and $CCR7^-Irf8^{hi}$ population frequencies. n=4-5 mice/group. **(D)** Expression of ICOS and CD69 on dLN (top) and tumor (bottom) DCs. n=4-5 mice per group. **(E)** Quantification of populations in (d) on dLN (left) and tumor (right). n= 4-5 mice/group. Data are shown as mean ± SEM. * p<0.05, **p<0.01, ***p<0.001. (2-way unpaired t-test).

Figure S6

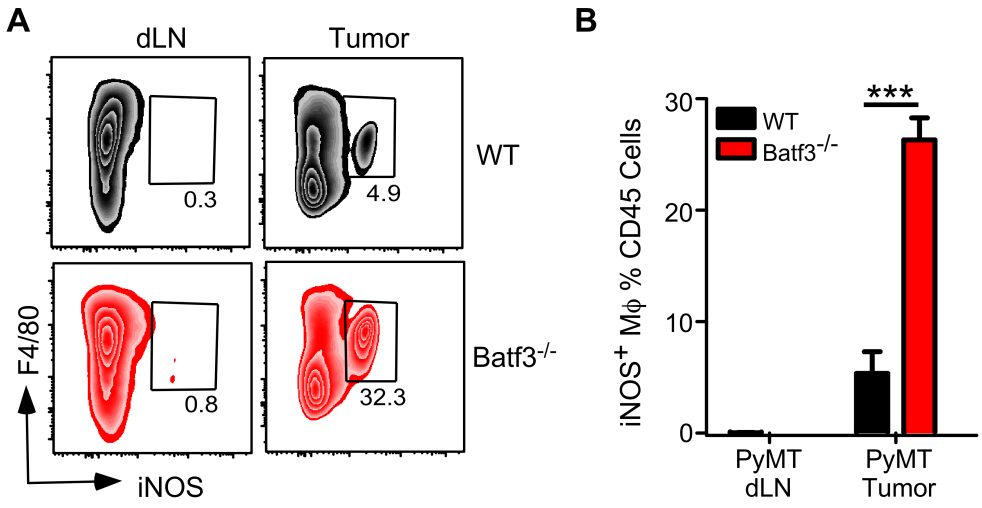

Figure S6. DC1 depletion causes the M1-Like polarization of TAMs in breast cancer. (A) Representative flow plots of iNOS and F4/80 on $CD11c^-$ $CD11b^+$ cells from the dLN (left) and the tumor (right) of mice with subcutaneous PyMT tumors at the endpoint. **(B)** Quantification of (a). n=4. Data are shown as mean ± SEM. ***p<0.001. (2-way unpaired t-test).

Figure S7

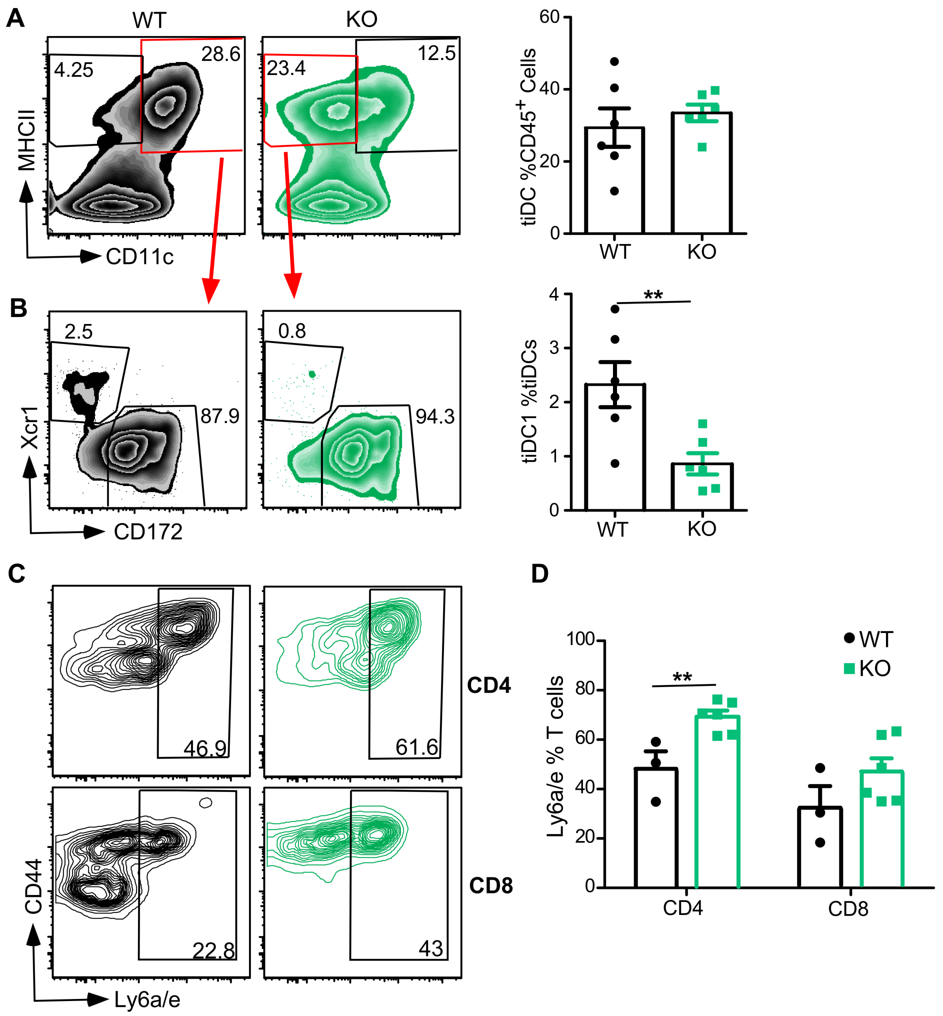

Figure S7. GP96 KODC display enhanced T cell priming and maintenance. (A) Representative flow plots of the frequency of tumor-infiltrating DCs gated on CD45^{+} B220^{-} cells from 18-week-old mice (left). Quantification of tiDCs (right). n=6. **(B)** Representative flow plots of the frequency of DC1 (Xcr1^{+}) and DC2 (CD172^{+}) within the indicated population from (a) (left). Quantification of tiDC1 (right). **(C)** Representative flow plots of CD44 and Ly6a/e expression on tumor-infiltrating CD4 and CD8 T cells from MMTV-PyMT mice at the endpoint. **(D)** Quantification of the frequency of Ly6a/e^{+} T cells from (c). n=3-6 mice per group. Data are shown as mean ± SEM. **p<0.01. (2-way unpaired t-test).
